# Editorial: Therapeutic targets and strategies for long COVID and post-viral syndrome

**DOI:** 10.3389/fcimb.2026.1820002

**Published:** 2026-03-13

**Authors:** Yajing Yang, Bowen Li, Jing Yang

**Affiliations:** 1 Department of Rheumatology and Immunology, Anqing Hospital, the Hospital of Anhui Medical University, Anqing, China; 2 Department of Hematology, Tongji Hospital, Frontier Science Center for Stem Cell Research, Shanghai Key Laboratory of Signaling and Disease Research, School of Life Sciences and Technology, Tongji University, Shanghai, China

**Keywords:** biomarkers, gut microbiota, immune regulation, long COVID, post-viral syndrome (PCS), T cell activation, therapeutic targets, vaccine prevention

## Introduction

Long COVID and post-viral syndrome (PCS) represent a persistent challenge to global public health, characterized by prolonged symptoms such as severe fatigue, cognitive impairment, and respiratory distress. As the global disease burden escalates, the traditional paradigm positioning these sequelae as mere prolonged recovery phases is proving inadequate. Emerging evidence reveals them as complex, context-dependent immunometabolic entities. Unraveling the precise molecular networks driving these enduring symptoms—from cellular exhaustion to microbial dysbiosis—has become crucial for advancing translational medicine and developing targeted clinical interventions. Despite extensive clinical observations, the underlying pathogenesis of Long COVID remains incompletely understood, leaving a significant gap in targeted diagnostic and therapeutic strategies. This Research Topic aims to address this critical need by bringing together multidisciplinary studies that explore potential therapeutic targets and effective intervention strategies. The collection of articles presented here investigates the syndrome from multiple perspectives, including gut microbiota metabolic alterations, immune regulation, broad-spectrum vaccine development, biomarker identification, and the longitudinal dynamics of T cell activation.

## Gut microbiota and metabolic alterations

In the context of understanding post-viral symptom persistence, the gut-immune axis has emerged as a critical area of investigation. Brigo et al. explored the metabolic alterations in Long COVID patients using advanced urine metabolomic analysis. They demonstrated that bacteria-related uremic metabolites, specifically 4-hydroxyphenylpropionic acid (HPHPA) and tryptamine, were significantly elevated. Notably, 64% of Long COVID patients exhibited elevated levels of at least one metabolite, with hippuric acid emerging as a unique marker. These metabolic patterns successfully distinguished Long COVID patients from healthy controls and those with myalgic encephalomyelitis/chronic fatigue syndrome (ME/CFS). Heatmap cluster analysis provided direct evidence for these distinct metabolic patterns. Furthermore, the authors discussed previous evidence from animal models involving fecal microbiota transplantation, suggesting that microbial metabolic disorders can induce systemic inflammation and neurocognitive symptoms. These findings highlight a functional link between gut microbiome dysbiosis and Long COVID, providing a potential metabolic basis for the syndrome.

## Immune regulation and synergistic effects with tumors

Systemic immune dysfunction in Long COVID can also complicate the clinical management of patients with co-morbidities, particularly malignancies. A comprehensive review by Lv et al. provided an overview of the immune characteristics in Long COVID patients and evaluated their impact on lung cancer immunotherapy. The authors detailed specific immune abnormalities, including T cell exhaustion, neutrophil extracellular trap (NET) formation, and the chronic activation of memory B cells. Interestingly, they highlighted clinical evidence suggesting that COVID-19 vaccination may improve the efficacy of immune checkpoint inhibitors in patients with non-small cell lung cancer (NSCLC), significantly improving both progression-free and overall survival in advanced stages. The review also discussed how related immune disorders might promote tumorigenesis through pathways such as the renin-angiotensin-aldosterone system (RAAS) and chronic inflammation-mediated cell transformation, offering a valuable perspective for cross-disease clinical management.

## Vaccine prevention and broad-spectrum protection

To address the continuous immune escape of SARS-CoV-2 variants, establishing robust preventive measures is essential. Chen et al. developed a broad-spectrum receptor-binding domain (RBD) vaccine candidate (M5-RBD) incorporating key mutation sites (K417T, L452R, T478K, E484K, and N501Y) and a novel CpG adjuvant HP007. Their rigorous animal experiments demonstrated that the vaccine induced durable T cell responses and broad-spectrum neutralizing antibodies against multiple variants, including the Omicron BA.2 strain. Antigenic cartography revealed significantly shortened antigenic distances against various variants. In K18-hACE2 knock-in mouse models, the authors showed that the vaccine effectively inhibited viral replication in the lungs, brain, and nasal turbinates, subsequently reducing histopathological damage. This study provides a promising framework for the design and development of next-generation vaccines.

## Biomarker identification via extracellular vesicles

In the search for reliable tools for precise disease monitoring, Fanelli et al. explored the diagnostic value of extracellular vesicles (EVs) circulating in patient plasma using flow cytometry and atomic force microscopy. They found that EVs expressing CD169+ and HLA-DR+ were persistently elevated in individuals with Long COVID. Consistent with ongoing systemic inflammation, these specific EVs correlated closely with abnormal coagulation function and elevated levels of inflammatory factors, such as IL-6 and TNF-alpha, as well as coagulation parameters like D-dimer. The researchers noted that these phenotypic changes were particularly evident in medium-to-large EVs (240–500 nm and >500 nm), correlating with monocyte surface markers and suggesting their active involvement in intercellular communication and immune activation. These results underscore the potential of using extracellular vesicles as integrated biomarkers to monitor both immune and coagulation abnormalities.

## Longitudinal dynamics of T cell activation

Moreover, longitudinal follow-up studies are indispensable for understanding the clinical course and immune recovery trajectories of Long COVID. Ueland et al. evaluated the plasma levels of specific T cell activation and exhaustion markers over an 18-month period in patients specifically recovering from mild COVID-19. They demonstrated that soluble markers, specifically sCD25, sTIM-3, and sLAG-3, were closely associated with the persistence of symptoms. Patients experiencing ongoing dyspnea and fatigue exhibited significantly higher sCD25 levels (60-74% higher during 6–18 months), while those reporting memory problems showed increased sLAG-3 levels. Additionally, a correlation between sCD25 and neutralizing antibody titers was identified exclusively in symptomatic patients. These findings suggest that distinct circulating markers accurately reflect different dimensions of immune dysregulation, providing a highly specific basis for targeted clinical assessment.

## Conclusion

Collectively, the studies compiled in this Research Topic underscore that Long COVID is not a singular prolonged illness, but rather a highly heterogeneous syndrome driven by intertwined metabolic and immunological disruptions. Rather than viewing these post-viral complications through a single mechanistic lens, the findings presented here highlight the necessity of a multi-dimensional approach. From the specific alterations in gut-derived metabolites to the longitudinal trajectories of T cell exhaustion, these distinct biological markers offer a clearer roadmap for precise patient stratification. Moving forward, the critical next step lies in translating these diverse biological targets into integrated clinical tools and personalized therapeutic regimens. We hope that the insights gathered in this Research Topic will inspire continued multidisciplinary collaboration, ultimately guiding the transition from generalized symptom management toward rationally designed, individualized care for patients burdened by Long COVID.

